# Longitudinal Analysis of Respiratory Function of Different Types of Limb Girdle Muscular Dystrophies Reveals Independent Trajectories

**DOI:** 10.1212/NXG.0000000000200084

**Published:** 2023-07-10

**Authors:** Robert Muni-Lofra, Eduard Juanola-Mayos, Marianela Schiava, Dionne Moat, Maha Elseed, Jassi Michel-Sodhi, Elizabeth Harris, Michelle McCallum, Ursula Moore, Mark Richardson, Christina Trainor, Karen Wong, Monika Malinova, Carla Bolano-Diaz, Michael John Keogh, Elisabetta Ghimenton, Jose Verdu-Diaz, Anna Mayhew, Michela Guglieri, Volker Straub, Meredith K. James, Chiara Marini-Bettolo, Jordi Diaz-Manera

**Affiliations:** From the John Walton Muscular Dystrophy Research Centre (R.M.-L., M.S., D.M., M.E., J.M.-S., E.H., M. McCallum, U.M., M.R., C.T., K.W., M. Malinova, C.B.-D., M.J.K., E.G., J.V.-D., A.M., M.G., V.S., M.K.J., C.M.-B., J.D.-M.), Translational and Clinical Research Institute, Newcastle University, UK; Highly Specialized Service for Rare Neuromuscular Disorders (R.M.-L., M.S., D.M., M.E., J.M.-S., E.H., M. McCallum, U.M., M.R., C.T., K.W., M. Malinova, C.B.-D., M.J.K., E.G., J.V.-D., A.M., M.G., V.S., M.K.J., C.M.-B., J.D.-M.), Limb Girdle Muscular Dystrophies, Genetics Department, Integrated Laboratory Medicine, Newcastle Upon Tyne Hospitals NHS Foundation Trust, United Kingdom; and Neuromuscular Diseases Unit, Neurology Department, Hospital Germans Tries I Pujol (E.J.-M.), Badalona, Spain.

## Abstract

**Background and Objectives:**

The prevalence and progression of respiratory muscle dysfunction in patients with limb girdle muscular dystrophies (LGMDs) has been only partially described to date. Most reports include cross-sectional data on a limited number of patients making it difficult to gain a wider perspective on respiratory involvement throughout the course of the disease and to compare the most prevalent LGMD subtypes.

**Methods:**

We reviewed the results of spirometry studies collected longitudinally in our cohort of patients in routine clinical visits from 2002 to 2020 along with additional clinical and genetic data. A linear mixed model was used to investigate the factors associated with the progression of respiratory dysfunction.

**Results:**

We followed up 156 patients with 5 different forms of LGMDs for a median of 8 years (range 1–25 years). Of them, 53 patients had pathogenic variants in the *Capn3* gene, 47 patients in the *Dysf* gene, 24 patients in the *Fkrp* gene, 19 in the *Ano5* gene, and 13 in one of the sarcoglycan genes (SCG). At baseline, 58 patients (37.1%) had a forced vital capacity percentage predicted (FVCpp) below 80%, while 14 patients (8.9%) had peak cough flow (PCF) values below 270 L/min. As a subgroup, *FKRP* was the group with a higher number of patients having FVC <80% and/or PCF <270 L/min at initial assessment (66%). We observed a progressive decline in FVCpp and PCF measurements over time, being age, use of wheelchair, and LGMD subtype independent factors associated with this decline. *Fkrp* and sarcoglycan patients had a quicker decline in their FVC (Kaplan–Meier curve, F test, *p* < 0.001 and *p* = 0.02, respectively). Only 7 of the 58 patients with low FVCpp values reported symptoms of respiratory dysfunction, which are commonly reported by patients with FVCpp below 50%–60%. The number of patients ventilated increased from 2 to 8 during follow-up.

**Discussion:**

Respiratory dysfunction is a frequent complication of patients with LGMDs that needs to be carefully studied and has direct implications in the care offered in daily clinics. Respiratory dysfunction is associated with disease progression because it is especially seen in patients who are full-time wheelchair users, being more frequent in patients with mutations in the *Fkrp* and sarcoglycan genes.

Limb girdle muscular dystrophies (LGMDs) are a group of genetic diseases characterized by progressive degeneration of skeletal muscles, leading to muscle weakness and disability. LGMDs are produced by mutations in genes codifying proteins with different locations and function in the muscle fibers. The list of genes causing LGMDs has been continuously growing since the description of the alpha-sarcoglycan gene (*Sgca*) in 1994, which was the first gene linked to an LGMD.^1^ According to the most recent classification, there are 32 genes causing LGMDs, 27 with a recessive inheritance and 5 with a dominant inheritance pattern.^2^ The epidemiology of LGMDs is not known; however, as a group, the estimated prevalence ranges from 1 in 14,500–45,000.^3,4^ The frequency of each LGMD subtype has also not been established, although recent studies suggest that the 5 most prevalent types are caused by pathogenic variants in the genes *Capn3* (calpainopathy or LGMD-R1), *Dysf* (dysferlinopathy or LGMD-R2), *Ano5* (anoctaminopathy or LGMD-R12), *Fkrp* (Fukutin-related protein myopathies or LGMD-R9), and the sarcoglycan genes: *Sgca* (alpha-sarcoglycanopathy*)*, *Sgcb* (beta-sarcoglycanopathy), or *Sgcg* (gamma*-*sarcoglycanopathy) or LGMD R3, 4, and 5, respectively.^5^

Patients with LGMD develop progressive muscle weakness in the shoulder and pelvic girdle associated with a variable degree of disability, ranging from patients with mild difficulties walking to patients who are full-time wheelchair users. Cardiac and respiratory muscles can also be affected in some of these conditions, as has been reported in previous singular cohorts of patients with pathogenic variants in the *Fkrp*,^6,7^ sarcoglycan,^8,9^ and *Capn3*^10,11^ genes. However, data on the progression of cardiac and respiratory dysfunction over time are lacking for most of these conditions. Identifying whether there are LGMD subtypes with an increased risk of developing cardiac and respiratory complications as well as understanding whether there are other clinical factors associated with this dysfunction could be useful to provide correct prognosis and arrange appropriate follow-up care. Moreover, to know whether and how respiratory function progresses over time in each subtype could be informative for clinical trial design. The main aim of this study was to study respiratory muscle function in a cohort of patients with LGMDs followed up in our clinics to identify whether there are subgroups of LGMD with the highest risk of respiratory insufficiency and to establish the progression of respiratory muscle function over time.

## Methods

### Study Population

We included all patients seen at the Highly Specialized Service for Limb Girdle Muscular Dystrophies in Newcastle upon Tyne with genetically confirmed LGMDs caused by pathogenic variants in one of the following genes: *Capn3, Dysf, Fkrp, Ano5, Sgca, Sgcb, Sgcg*. Patients with pathogenic variants in the sarcoglycan genes (*Sgca, Sgcb*, and *Sgcg*) are studied together due to the low number of patients by subgroup and because there are previous data suggesting that these patients do not show differences in respiratory dysfunction^12^ when compared among them. Data were prospectively collected in our clinics from April 2002 to October 2020. Patients were seen every 12–18 months or more frequently, based on clinical judgment.

### Standard Protocol Approvals

The study was approved as a service review under the Newcastle upon Tyne Trust Hospital because the project was to evaluate the current natural history data to inform decision-making process on respiratory management.

### Respiratory Function

Spirometry was performed as part of the standard clinical assessment using a Microlab Spirometer (ML3500MK8) at each visit by trained physiotherapists with extensive experience in pulmonary function testing. The best of at least 3 efforts deemed reliable by the physiotherapist was recorded. Force volume capacity (FVC) was measured in sitting and lying positions when possible. A cutoff point of 80% to define an FVCpp as pathologic was selected based on reference values used in previous publications.^10,13^ Peak cough flow (PCF) was also measured according to clinical criteria. A cutoff point of 270 L/min to define a PCF as pathologic was selected based on the recommendations of the British Thoracic Society.^14^ For all these parameters, raw values were collected for the purpose of this study together with height, age, and ethnic background to obtain FVC predicted values (FVCpp) according to the Global Lung Initiative.^15^ Diaphragmatic dysfunction was considered if FVCpp dropped at least 10% when patients were assessed lying compared with that when sitting aligned with similar criteria used in previous publications.^14^ Ventilation requirement, either noninvasive (NIV) or invasive (tracheostomy), and comorbidities (smoker/nonsmoker, asthma or other respiratory diseases) were recorded at each visit. The presence of symptoms suggestive of respiratory dysfunction were carefully assessed at each visit, including dyspnea at rest or after exercise and symptoms suggestive of nocturnal hypoventilation.

### Statistical Analysis

Data were expressed as numbers and percentages for categorical variables and as mean ± SD or median and interquartile range (IQR) for quantitative variables. The Kolmogorov-Smirnov test was used to determine the normality of distribution of quantitative variables. Time of disease duration at first assessment was defined as time in years from onset of symptoms as reported by the patient/relative to the neuromuscular team at their first clinical visit. Follow-up time was defined as the time in years from the first clinical visit to the most recent clinical visit with the neuromuscular team.

The χ^2^ test was used to compare dichotomous variables between patients with FVC higher and lower than 80% including gender, wheelchair use, smoker status, and the presence of other respiratory comorbidities. To compare continuous variables between groups, such as FVCpp or PCF, we used ANCOVA with age as a confounding variable and run paired comparisons between all LGMDs to identify significant differences.

A multilevel mixed-effect model with random intercepts and random slopes, which accounts for repeated measurements on the same patient, was used to explore the relationship between the LGMD type and the FVCpp and between the LGMD type and the PCF (L/min). Patients who had fewer than 2 follow-up values in the FVCpp were excluded from the analysis. For the FVCpp analysis, fixed effects were the intercepts, LGMD type, time (age in years since born), and LGMD type-time interaction. For the PCF (L/min) analysis, fixed effects were the intercepts, LGMD type, time (age and age square), and LGMD type-time interactions. Random effects were time age and intercepts for subjects. An unstructured covariance pattern was used in all the models. Convergence was achieved in both models. F tests were used to test whether the mean FVCpp trajectories differed by LGMD type using ANO5 as reference group.

A time-to-event analysis was performed to determine the time to a sitting FVCpp below 80% and the time to a PCF absolute value below 270 L/min. The median age at events and the corresponding 95% confidence intervals (CIs) were estimated by plotting Kaplan-Meier curves for participant groups defined by the type of LMGD.

Using receiver operating characteristic (ROC) curves were performed to identify a cutoff point of the FVCpp sitting that could identify patients who had symptoms of respiratory failure or the need of ventilatory support. A level of significance of 0.05 was used for hypothesis testing. Statistical analysis was performed using IBM SPSS statistics, version 27. Graphs were performed using either SPSS or R software version 4.2.1.

### Data Availability Statement

The authors confirm that aggregated data are available under reasonable request to the corresponding authors.

## Results

### Description of the Cohort

We included 156 patients with genetically confirmed LGMD (Table 1). The median age at first visit was 37 (IQR: 26–50) years, with variation between LGMD subtypes (Table 1). The median time of disease duration at first assessment was 21 years (IQR: 5–48). The median follow-up time was 8 years (IQR 1–16 years), and the median number of visits per patient during this period was 4 (IQR 1–6 visits), for a total of 601 visits.

**Table 1:**
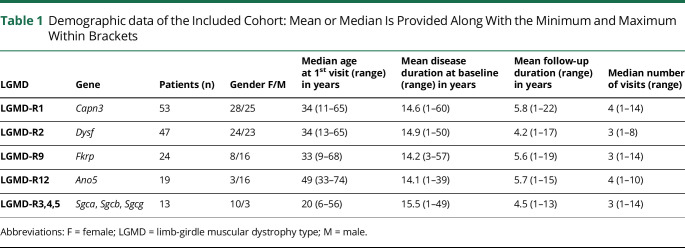
Demographic data of the Included Cohort: Mean or Median Is Provided Along With the Minimum and Maximum Within Brackets

At baseline, 24 (15.3%) patients were nonambulant, and only 2 (1.2%) required noninvasive ventilatory support using BiPAP for nocturnal hypoventilation. Ten (6.4%) patients had concomitant respiratory conditions, more commonly asthma (n = 5) and sleep apnea (n = 2).

FVCpp and PCF at baseline and last visit for the entire cohort and each LGMD subtype are reported in eFigure 1 (links.lww.com/NXG/A617). The median FVCpp was <80% only in the patients with pathogenic variants in *Fkrp* at baseline, which was significantly lower than the other 4 groups (eFigure 1A). A total of 58 patients (37.1%) at baseline visit had FVCpp <80%: 15 *Capn3* patients, (28.3% of total *Capn3*), 21 *Dysf* patients (44.6% of total *Dysf*), 16 *Fkrp* patients (66% of total *Fkrp*), 2 *Ano5* patients (10.5% of total *Ano5*), and 4 sarcoglycan patients (30.7% of total sarcoglycans). Of these 58 patients, 16 (27.6%) were nonambulant, while 7/58 (12.1%) reported symptoms of respiratory dysfunction. A difference in FVCpp between lying and sitting >10%, suggestive of diaphragm dysfunction, was observed in 11/156 patients (7.0%) at baseline. However, this increased to 16/156 (10.3%) at the last visit, being more commonly observed in *Fkrp* patients during follow-up (eFigure 1E). PCF values also declined over time (eFigure 1, C and F). Fourteen (9.0%) patients had a PCF <270 L/min at baseline, while at the last visit, PCF values declined in 9 more patients to fall under PCF <270 L/min value for a total of 23 patients at last visit (14.7%). All *Ano5* patients had a PCF above 270 L/min during the follow-up. At the last visit, 8 (5.1%) patients required NIV, 2 *Capn3* patients, 2 *Dysf*, 3 *Fkrp*, and 1 sarcoglycan patient. None of the patients who required NIV had other concomitant respiratory conditions, except 1 who had a sleep apnea associated. Of interest 4 of the 8 patients on NIV had a confirmed drop in FVCpp of more than 10%, and in 2 more, FVC was not tested when lying because patients did not tolerate that position.

### Progression of FVCpp Over the Follow-up Period

One hundred four patients had follow-up visits at our center. The last visit took place at a median of 8 years (range 1–25 years) after the first appointment with a median of 4 follow-up visits during that period. Demographic and clinical factors associated with FVCpp <80% by LGMD subgroup are reported in Table 2.

**Table 2:**
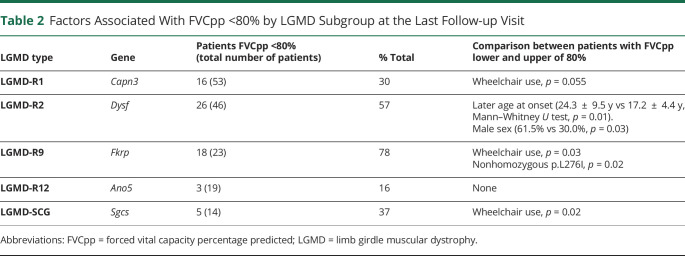
Factors Associated With FVCpp <80% by LGMD Subgroup at the Last Follow-up Visit

**Figure 1:**
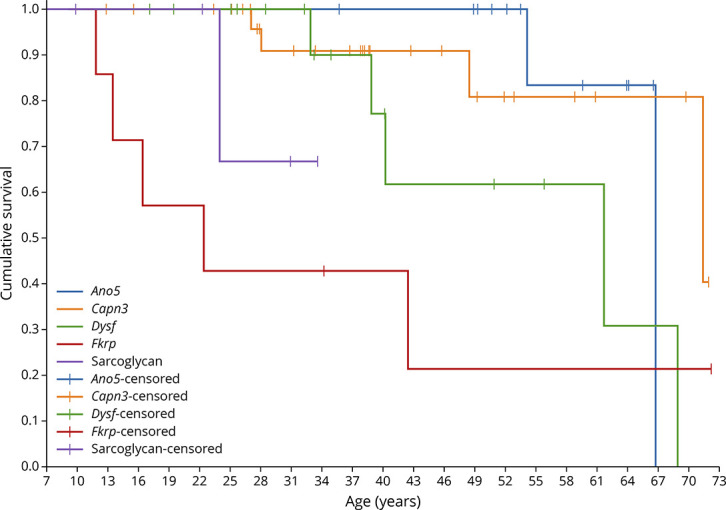
Kaplan-Meier Estimator for a Forced Vital Capacity Percentage Predicted Lower Than 80% by LGMD Kaplan–Meier curves showing age and number of patients whose forced vital capacity percentage predicted values dropped below 80% during the follow-up. LGMD = limb girdle muscular dystrophy.

Of the 104 patients with follow-up data, 36 (34.6%) had an FVCpp below 80% during the first assessment, which persisted to the last follow-up. These 36 patients were excluded from the survival analysis. Among the remaining 69 patients (*Capn3*, 29; *Dysf*, 16; *Fkrp*, 7; *Ano5*, 12; and LGMD-SCG, 5), significant differences in the age at which FVCpp fell less than 80% were observed by LGMD type (Log rank Mantel-Cox χ^2^ 11.34 *p* = 0.023, Breslow χ^2^ 30.14 *p* < 0.01, and Tarone-Ware χ^2^ 24.06 *p* < 0.01) (Figure 1). The median values for survival time for FVC higher than 80% by LGMD are summarized in eTable 1 (links.lww.com/NXG/A617).

**Figure 2:**
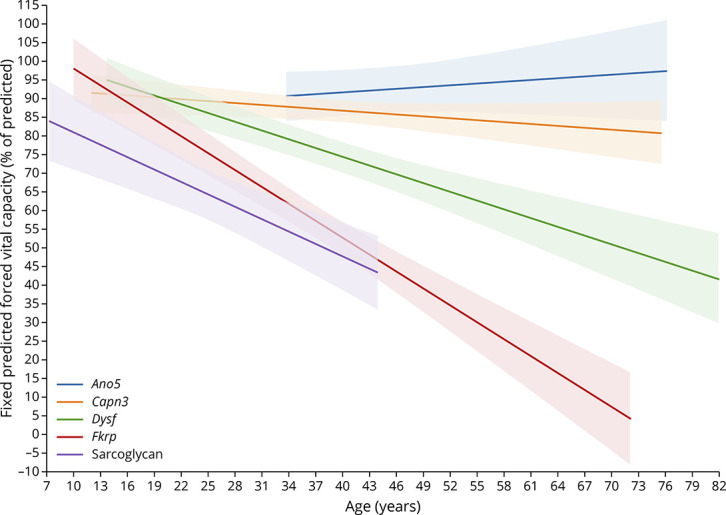
Progression of Forced Vital Capacity Percentage Predicted Values During the Follow-up The graph shows forced vital capacity percentage predicted values for all patients included in the study over time plotted by the age of the patients. Bold line shows the mean value at each time point, and the shaded area shows the SD.

The sitting FVCpp by LGMD subtype followed a linear trajectory and is shown in Figure 2 and eFigure 2 (links.lww.com/NXG/A617). The age of the patients and the interaction between age and the type of LGMD were significant predictors of the FVCpp trajectories (age and age-LGMD type interaction mixed-effect model *p* < 0.01 for both fixed effects over time). The mean FVCpp by LGMD type and by decade of life is summarized in eTable 2. Mean FVCpp trajectories differed significantly between the LGMD subgroups, showing *Fkrp* and LGMD-SCG patients an earlier and more frequent decrease of FVCpp less than 80% (F test, *p* < 0.001 and *p* = 0.02, respectively) as summarized in eTable 3. *Capn3* and *Ano5* patients showed estimated mean FVCpp higher than 80% during the whole study period and in all decades. Patients with mutations in the *Capn3, Dysf, Fkrp*, and sarcolgycan genes showed a yearly decline in FVC, whereas *Ano5* patients were stable over time (Figure 2). In the linear model, *Fkrp* and LGMD-SCG patients showed the greatest decline, losing 1.45 (SE 0.53) units and 1.55 (SE 0.55) units of FVCpp per year, respectively (Table 3).

**Table 3:**
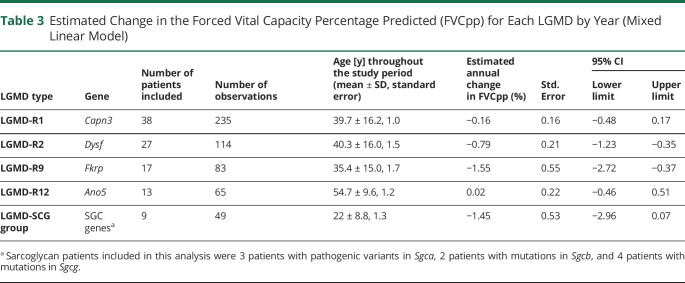
Estimated Change in the Forced Vital Capacity Percentage Predicted (FVCpp) for Each LGMD by Year (Mixed Linear Model)

### Progression of PCF Over the Follow-up Period

**Figure 3:**
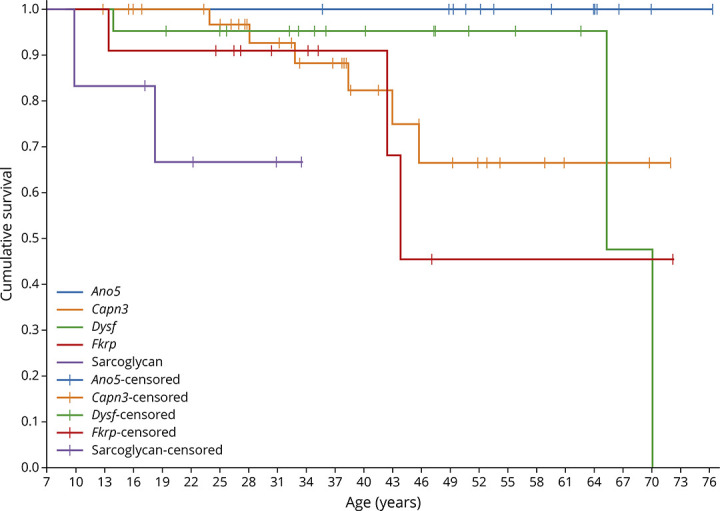
Kaplan–Meier Estimator for a Peak Cough Flow Lower Than 270 L/min by LGMD Kaplan–Meier curves showing age and number of patients whose peak cough flow value dropped lower than 270 L/min during the follow-up. LGMD = limb girdle muscular dystrophy.

Similar to FVCpp, PCF values also declined during the follow-up period. Twenty-one of the 156 original patients were excluded from the PCF survival analysis: 14 of 135 remaining already had a PCF <270 L/min at baseline, staying below that threshold at the last follow-up (*4 Capn3, 5 Fkrp*, and 5 in LGMD-SCG), and in the remaining 7 patients, the PCF value was not available. Time to PCF falling <270 L/min was significantly different by LGMD type (Figure 3). All *Ano5* patients remained with a PCF above 270 L/min at the last assessment. Patients with LGMD-SCG showed an earlier fall of PCF value < 270 L/min. The mean age when PCF fell <270 L/min by LGMD type is summarized in eTable 3 (links.lww.com/NXG/A617).

**Figure 4:**
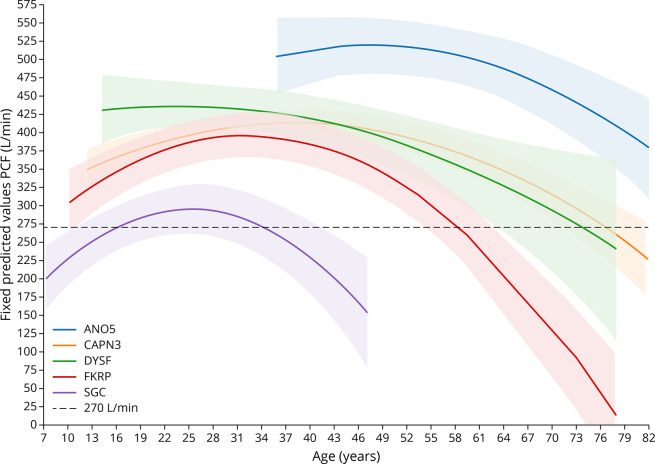
Progression of Peak Cough Flow Percentage Predicted Values During the Follow-up The graph shows Peak Cough Flow percentage predicted values for all patients included in the study over time plotted by the age of the patients. Bold line shows the mean value at each time point, and the shaded area shows the SD.

PCF in all LGMD subtypes followed a quadratic trajectory, which are shown in Figure 4 and eFigure 3 (links.lww.com/NXG/A617). Age was a significant predictor of the PCF absolute value (age and age square, mixed-effect model *p* < 0.05). *Ano5* patients were the only group who showed predicted PCF higher than 270 L/min. Patients with *Capn3, Dysf, Fkrp,* and LGMD-SCG showed a decline in the PCF, whereas *Ano5* patients were stable (Figure 4).

### Respiratory Symptoms and Need for Ventilation

Using ROC curves we aimed to identify a cutoff point of the FVCpp sitting that could identify patients who had symptoms of respiratory failure, such as effort dyspnea or orthopnea, or the need of ventilatory support. In both cases, the curves obtained had a very low area under the curve (AUC) (AUC 0.16 and AUC 0.04, respectively) suggesting that FVCpp cannot predict respiratory symptoms or the need of ventilation with either high sensitivity or specificity (eFigure 2, links.lww.com/NXG/A617).

## Discussion

In this analysis of 156 patients with LGMDs followed up for a mean of 8.3 years, using standardized spirometry and detailed clinical assessment, we observed significant differences in spirometry values at baseline and in their trajectories over time between the different LGMD groups. Patients with pathogenic variants in the *Capn3, Dysf, Fkrp*, and the sarcoglycan genes demonstrated a decline in FVC over time, with the FVCpp declining faster for *Fkrp* and LGMD-SCG patients and slower for *Capn3* and *Dysf* patients. The FVCpp trajectories for *Ano5* patients remained unchanged and > 80% FVCpp over time.

There are only a few published studies assessing respiratory muscle function using spirometry in patients with LGMD^6–11^ and with limited analysis of change over time. Our results are consistent with much of the published literature. In our cohort, *Fkrp* group had the largest proportion of patients (66%) with FVCpp <80% at baseline, a value that has been classically considered as indicative of respiratory dysfunction but also used in previous similar publications.^10,13^ In addition, *Fkrp* was the group with the most significant progression during follow-up, with an annual decline of 1.55% FVCpp. A previous cross-sectional study of a cohort of 36 *Fkrp* patients showed that 36% had moderate respiratory impairment, defined as FVCpp 41%–75%, while 8% had severe impairment (defined as FVCpp 19%–40%), which is in agreement with what we have shown in this study.^6^ Similarly, data collected from the *Fkrp* registry revealed that 15% of patients used NIV at an average age of 40 ± 15 years, being patients homozygous for the Leu276Ileu mutation less severely involved, as we have observed in our cohort.^16^

For LGMD-SCG patients, pathologic FVCpp values have been reported in 76%–86%, with no significant difference among the genetic subtypes.^8,9^ A large study of 439 *SCG* patients observed that up to 25% required NIV at a mean age of 29.1 years (range 8–58),^12,17^ with no significant differences among the different sarcoglycan gene mutations. Our study identified that although many LGMD-SCG patients had FVCpp higher than 80% at baseline, most had PCF values already <270 L/min, suggesting that this group of patients might have early difficulties removing secretions and may potentially benefit from cough augmentation interventions. However, the percentage of *SCG* patients requiring NIV in our cohort was lower than the percentage that was published earlier. Respiratory dysfunction progressed for LGMD-SCG with an annual loss of 1.45% FVCpp, in accordance with recently published data from the Italian registry without differences based on the sarcoglycan gene mutated.^18^ In the case of *Dysf* patients, a recent longitudinal study showed that, although only 24% of 182 patients had FVCpp values <80% at baseline, FVCpp worsened slowly over time with only an additional 4% dropping below 80% over 3 years of follow-up.^11^ These data were similar to previous publications that identified just a minority of *Dysf* patients with FVCpp values lower than 80%.^10,19^ Contrasting with these results, 48% patients in a subgroup of the Japanese cohort of patients with mutations in the *Dysf* gene with late onset had FVCpp values lower than 80%.^13^ The case of *Capn3* is especially intriguing because we observed 2 different trajectories. A subgroup of patients presented with normal values that did not progress significantly over time, while approximately 15% of the patients presented with low FVCpp values at baseline and/or clearly progressed during the follow-up, as previously reported in other cohorts.^20^ We did not find any clinical difference between these groups, although a higher, but nonsignificant, proportion of those who progressed were full-time wheelchair users, suggesting that respiratory involvement could follow limb muscle weakness.^21^ Another study reported FVC in a cohort of 85 *Capn3* patients and identified only 10 (11.8%) with FVCpp below <50% and only 1 patient requiring NIV.^20^ Our *Ano5* cohort showed normal values over the whole follow-up period. However, we did observe a slowly but constant reduction in FVCpp over time, which could lead to pathologic values after years of progression. This slower progression is consistent with previous reports of lack of respiratory involvement in this group, although longitudinal data have not been consistently collected in this population.^22^

We identified LGMD subgroup, the age of the patient at each assessment, and ambulatory status as factors significantly associated with progressive deterioration in lung function. Because only a few patients reported active smoking or comorbid respiratory conditions, we were therefore not able to determine the influence of these 2 factors on the progression of respiratory involvement. Previous publications have identified specific genetic mutations to be associated with a more severe phenotype inclusive of respiratory involvement. For example, patients with homozygous truncating variants in any of the sarcoglycan genes have a quicker progression.^9,17^ For *Fkrp*, patients homozygous for the Leu276Ileu variant are less severely affected and are less likely to develop respiratory problems.^16^ The presence of scoliosis, a sign of axial muscle weakness during childhood, has been also associated with respiratory impairment.^12,17^

In our study, neither of the spirometry parameters could predict the presence of respiratory symptoms or need for ventilation with high sensitivity and specificity. In fact, despite 67 patients having FVCpp values <80%, only 14 had symptoms of respiratory dysfunction, such as breathlessness. This suggests that LGMD patients may not experience significant respiratory symptoms despite reduced lung function. A possible reason for this could be that the progression of generalized muscle weakness leads to lower levels of physical activity and hence reduced cardiorespiratory demand for these patients. Of interest, just a minority of patients showed clear evidence of diaphragmatic weakness in the spirometry, which could be one of the reasons why patients did not offer complain about orthopnea. Based on these data, we propose that respiratory function should be assessed routinely in all LGMD patients despite the absence of respiratory symptoms, at least every 3 to 5 years. If patients develop symptoms or have FVCpp lower than 80%, we support a more frequent assessment, probably every year. Based in our data, only *Ano5* patients could be excluded from a regular assessment based on our results in the absence of clinical data suggesting respiratory involvement.

Our study has several limitations. We designed and performed the analysis retrospectively. However, the assessments performed in our clinics at each visit are standardized, and spirometry is part of the regular tests although we do not have PCF for all patients assessed in our clinics. Because this is a monocentric study, the data presented in this study require validation in other cohorts followed in more centers. Being the LGMD included here autosomal recessive conditions, patients are generally assessed since the time of first clinical symptom/concern and not during presymptomatic stages. This implies that data regarding respiratory function before the first clinical assessment are not available, which leads to the exclusion of a considerable number of patients from the survival analysis because their FVCpp% was below 80% by the time of the first assessment. Not all patients had annual follow-up visits, with a few being seen only once before being referred to their local hospital although many patients were followed up for more than 10 years with several visits during this period. Another limitation is the low number of cases for some of the LGMD groups, especially for the LGMD-SCG patients (n = 13). In addition, we were not able to analyze the impact of body mass index on respiratory function because weight data were frequently missing. This could be a major contributor and should be considered in future studies.

In summary, this study characterizes the longitudinal progression of respiratory function in a large cohort of 5 subtypes of LGMD. We were able to identify risk factors for developing respiratory failure, which could inform guidelines for clinical care and suggest including respiratory measurements as an outcome measure for clinical trials in LGMDs.

## Glossary

AUC: area under the curve
CI: confidence interval
FVCpp: forced vital capacity percentage predicted
IQR: interquartile range
LGMD: limb girdle muscular dystrophy
NIV: noninvasive
PCF: peak cough flow
ROC: receiver operating characteristic
SCG: sarcoglycan genes
